# Apparently Resistant Hypertension in Polish Hemodialyzed Population: Prevalence and Risk Factors

**DOI:** 10.3390/jcm12165407

**Published:** 2023-08-20

**Authors:** Bartosz Symonides, Jacek Lewandowski, Wojciech Marcinkowski, Jacek Zawierucha, Tomasz Prystacki, Jolanta Małyszko

**Affiliations:** 1 Department of Internal Medicine, Hypertension and Vascular Diseases, Medical University of Warsaw, 02-091 Warsaw, Poland; bartosz.symonides@wum.edu.pl (B.S.); jacek.lewandowski@wum.edu.pl (J.L.); 2 Fresenius Medical Care, 60-118 Poznań, Poland; wojciech.marcinkowski@fmc-ag.com (W.M.); jacek.zawierucha@fmc-ag.com (J.Z.); tomasz.prystacki@fmc-ag.com (T.P.); 3 Department of Nephrology, Dialysis and Internal Diseases, Medical University of Warsaw, 02-097 Warsaw, Poland

**Keywords:** hypertension, resistant hypertension, hemodialysis, apparent treatment-resistant hypertension, end-stage renal disease

## Abstract

Background: The aim of this study was to assess the prevalence, characteristics, and determinants of apparent treatment-resistant hypertension (aTRH) in an unselected large population of patients with end-stage kidney disease (ESKD) treated with hemodialysis (HD) throughout the country. Methods: A database of 5879 patients (mean age 65.2 ± 14.2 years, 60% of males receiving hemodialysis) was obtained from the biggest provider of hemodialysis in the country. Hypertension and aTRH were defined using pre- or/and post-dialysis BP values. Patients with and without aTRH (non-aTRH) were compared. Results: Using pre- and post-dialysis criteria, hypertension was diagnosed in 90.7% and 89.1% of subjects, respectively. According to pre- and post-dialysis blood pressure criteria, aTRH incidences were 40.9% and 38.4%, respectively. The hypertensive patients with aTRH versus non-aTRH were younger, had a higher rate of cardiovascular disease, lower dialysis vintage, shorter time on dialysis, higher eKt/V, higher ultrafiltration, higher pre- and post-dialysis BP and HR, and higher use of antihypertensive drugs. Factors that increase the risk of aTRH according to both pre- and post-dialysis BP criteria were age—OR 0.99 [0.98–0.99] and 0.99 [0.98–0.99], the history of CVD 1.26 [1.08–1.46] and 1.30 [1.12–1.51], and diabetes 1.26 [1.08–1.47] and 1.28 [1.09–1.49], adjusted OR with 95% CI. Conclusions: In the real-life world, as much as 40% of HD patients may have aTRH. In ESKD HD patients, aTRH seems to be multifactorial, influenced by patient-related rather than dialysis-related factors. Various definitions of aTRH preclude easy comparisons between studies.

## 1. Introduction

**What is already known about this subject**

Hypertension is the most common finding in hemodialyzed patients; however, data based on large cohorts are limited.

The currently established definitions of resistant hypertension are not directly applicable to the end-stage kidney disease setting.

Apparent true resistant hypertension prevalence is unknown, and target blood pressure in patients on hemodialysis is still a matter of debate.

**What this study adds**

Apparent treatment-resistant hypertension, defined as uncontrolled blood pressure on three or more antihypertensive medication classes or the use of four or more medications regardless of blood pressure level, affects as much as 40% of HD patients in real-world settings.

**What impact this may have on practice or policy**

Determination of how and when blood pressure should be measured in the group of dialysis patients.

Revision of target blood pressure values in this group of patients is to be considered, as well as the definition of resistant hypertension or, rather, switch to apparent treatment-resistant hypertension is to be recommended.

Apparent treatment-resistant hypertension seems to be multifactorial, influenced by patient-related rather than dialysis-related factors; therefore, using standardized procedures may help to manage it.

Hypertension (HTN) affects the great majority of end-stage kidney disease (ESKD) on hemodialysis (HD), with a prevalence ranging from 82 to 89% depending on the criteria and technique of blood pressure (BP) measurement [1,2,3].

According to the current guidelines, treatment-resistant HTN (TRH) is defined in the general population as uncontrolled blood pressure on three or more antihypertensive drugs in adequate doses or when patients on four or more antihypertensive drug categories irrespective of the BP control, providing that antihypertensive treatment included diuretics [4,5].

True TRH occurs in 10–20% of people with hypertension [6,7]. Demographic and clinical characteristics indicate that patients with TRH, compared with the general population of patients with HTN, are older and more likely with comorbidities such as chronic coronary syndrome, chronic cardiac and renal insufficiency, migraine, cardiac and renal insufficiency, atrial fibrillation, diabetes, potency disorders, stroke, potency disorders, stroke or episode of transient central nervous system ischemia, and metabolic syndrome [8,9]. Numerous studies have shown that patients with RH are more likely to develop vascular complications (including stroke) and heart failure exacerbations requiring hospitalization [9]. In a large registry of patients with REACH atherosclerosis among more than 53,000 people with hypertension, TRH was present in 12.7% of patients. After a four-year follow-up, a multivariate analysis found that a composite endpoint (cardiovascular death, nonfatal fatal stroke, and myocardial infarction) occurred significantly more often in the resistant hypertension group than in the patients without resistant hypertension [10].

The currently established definitions of resistant hypertension are not directly applicable to the ESKD setting [11]. The diagnosis of true resistant hypertension requires confirmation of adherence to therapy and confirmation of uncontrolled BP values by ambulatory BP measurement (ABPM) or home BP measurement (HBPM) [5].

Since most epidemiological studies lack the key elements (medication doses, adherence to the therapy, etiology of HTN, ABPM measurement) to define true resistant hypertension, an additional category of HTN was proposed—namely, apparent treatment-resistant hypertension. According to American Heart Association, apparent treatment-resistant hypertension (aTRH) is defined as uncontrolled blood pressure with concurrent use of three antihypertensive medication classes or four or more antihypertensive medication classes regardless of blood pressure level when pseudoresistance cannot be excluded [4].

The prevalence of aTRH in the general population of patients with hypertension is estimated to be 0.5–14% and 1.6–42% in pre-dialysis chronic kidney disease (CKD) patients [12,13,14,15]. The prevalence of aTRH in CKD increases both with decreased GFR and raised albuminuria [12] and was shown to be significantly associated with increased cardiovascular risk in HD patients [16].

In our recent review [17], we discussed that the currently established definitions of resistant hypertension are not directly applicable to the end-stage renal disease setting. Additionally, we stressed that the target blood pressure values in this group of patients should be established. The definition of resistant hypertension in this group should be revisited, and its relationship to both subclinical and clinical endpoints should be established. We also underly that the studies assessing the incidence of aTRH in patients with ESKD on renal replacement therapy are scarce [17] and performed in various populations on different continents [11,16,18,19]. Moreover, these studies were performed in selected, usually tertiary centers; hence, their populations may not reflect real-world data.

Therefore, the aim of this study was to assess the prevalence, characteristics, and determinants of aTRH in an unselected large population of patients with ESKD treated with HD throughout the country.

## 2. Material

We performed a retrospective analysis of a fully anonymized database of 5879 unselected, consecutive prevalent patients (mean age 65.2 ± 14.2 years, 60% males) receiving hemodialysis from the biggest provider of hemodialysis in non-tertiary centers in Poland. The database included demographic data, the etiology of CKD, data on concomitant diseases and antihypertensive medication groups, pre- and post-dialysis BP and HR, and data regarding HD. Patient history and physical examination were generally used to assess the optimal target dry weight. The patients were questioned about interdialytic symptoms that suggested orthostatic hypotension (such as lightheadedness) and intradialytic symptoms such as muscle cramps. The reduction in dry weight was performed gradually. The target weight was reduced over days to weeks; the older the patient, the longer period of time was needed to achieve dry weight (in some cases up to 12 weeks). The target dry weight was reduced by 0.5 L per session. In patients who are unable to tolerate this, 0.2 L per session was attempted. If increasing the dialysis time was ineffective, the dialysate sodium concentration was reduced. The dialysate sodium was reduced gradually (i.e., 1 mEq/L every three to four weeks) to approximately 136 mEq/L. To assess volume status, body composition has been checked every 12–13 weeks by using a bioimpedance measurement device body composition monitor (BCM) as required by dialysis unit rules.

We analyzed the database updated on 6 October 2022, which included averaged data from a full 4-week period from 29 August 2022 to 25 September 2022.

The characteristics of the patients are depicted in Table 1.

## 3. Methods

BP measurements were performed by a dialysis nurse using a free-standing BP cuff in the sitting position according to the current guidelines using only sphygmomanometers that were certified and periodically validated by hospital technical authorities. We define hypertension and aTRH in each subject using two separate BP criteria, pre- or post-dialysis BP values. The normotension was defined as BP < 140/90 mm Hg in patients not using antihypertensive treatment. Hypertension was defined as BP ≥ 140/90 mm Hg and/or using antihypertensive medication.

Apparent treatment-resistant hypertension was defined as BP ≥ 140/90 while on 3 or more antihypertensive drugs or using 4 antihypertensive drugs, irrespective of BP values.

We adopted the definition used for the general population of hypertensives. This definition does not include volume status.

We analyzed the frequency of hypertension in the studied population according to these above-mentioned criteria. In patients with hypertension, we assessed the incidence of aTRH using pre- and post-dialysis BP values. We compared the hypertensive patients with aTRH and without aTRH (non-aTRH). The comparison included demographic data (age, gender), BMI, data on concomitant diseases (Charlson comorbidity index score, congestive heart failure, coronary artery disease, peripheral artery disease, cerebrovascular disease, diabetes mellitus, pre-dialysis sodium, pre- and post-dialysis BP and HR, and data regarding HD (dialysis vintage, effective weekly dialysis time, Kt/v, and ultrafiltration). Cardiovascular disease (CVD) was defined as the diagnosis of at least one of the following: heart failure, coronary artery disease, peripheral artery disease, and cerebrovascular disease.

### 3.1. Statistical Analysis

Group differences in continuous variables were calculated using the *t*-test. Categorical variables were compared using the chi-square test.

We calculated unadjusted and adjusted odds ratios with 95% CI for risk factors for the presence of aTRH that included age, sex, BMI, CVD history, diabetes mellitus, dialysis vintage, effective weekly treatment time, eKt/V, and ultrafiltration.

Risk estimates were calculated using a multivariable logistic regression model with binary outcomes for aTRH. The statistical analysis of the data was performed using R (R version 4.1.2, R-core Team, R Foundation for Statistical Computing, Vienna, Austria, 2021, https://www.r-project.org) (accessed on 1 August 2023).

A two-tailed *p*-value less than 0.05 was considered statistically significant.

### 3.2. Ethics

At the moment when a patient starts renal replacement therapy, she/he signs the written informed consent and additional consent that she/he is to be informed when his or her personal data—even anonymized data—are collected in a database and transferred to the health care provider. Anytime she/he has a right to withdraw the consent. Ethical approval was waived by the local Ethics Committee since the database was collected for the use of a healthcare provider, and all data were fully anonymized before the authors had any access to them. Moreover, there was no direct patient contact whatsoever.

## 4. Results

The prespecified criteria for hypertension were fulfilled by 5330 pts. (90.7%) according to pre-dialysis BP criteria and by 5240 pts. (89.1%) according to post-dialysis BP criteria. The characteristics of all patients, and those with/without hypertension, are presented in Table 1. The data regarding the etiology of renal disease were provided in 2231 pts. (37.9%) of all patients, with diabetic nephropathy being the most common (30%). The other established etiology included glomerulonephritis (22%), vascular disease or hypertension (21%), cystic kidney disease (15%), and chronic pyelonephritis (13%).

The incidence of aTRH according to pre-dialysis BP criteria was 40.9%, and 38.4% according to post-dialysis BP criteria. The hypertensive patients with aTRH compared with the non-aTRH group were younger and more frequently men; had higher BMI and Charlson comorbidity scores; higher frequency of CVD, congestive heart failure, cerebrovascular disease, and diabetes; lower dialysis vintage; shorter time on dialysis; higher eKt/V; higher ultrafiltration rates; higher pre- and post-dialysis BP values; lower pre- and post-dialysis heart rates; and higher use of antihypertensive drugs disregarding the criteria of aTRH used. The comparison of these groups is depicted in Table 2 and Table 3.

However, the multivariate analysis revealed that the only significant, independent factors that increase the risk of aTRH were lower age, higher BMI (pre-dialysis BP criteria only), a history of CVD, and diabetes (Table 4).

## 5. Discussion

It has been reported that over 50 to 60 percent of hemodialysis patients (up to 85 percent in some reports) and nearly 30 percent of peritoneal dialysis patients are hypertensive [2,19,20,21]. In one multicenter trial on 2535 adult HD patients, the prevalence of hypertension, defined as one-week average pre-dialysis systolic BP measurements of 150 mmHg or diastolic BP 85 mmHg or the use of antihypertensive medications, was 86 percent [3,4]. However, there is a scarcity of new epidemiological data on hypertension prevalence in the HD population; in particular, there is very limited data on resistant hypertension in these patients. Our study showed the high prevalence of aTRH ranging from 38.4% to 40.9% in a large, unselected population of patients with ESKD treated with HD throughout the country. It should be stressed that this is a large and representative sample, and this sample’s size and representativeness enhance the generalizability of the findings to a broader ESKD population receiving HD.

In the classical study performed on 369 HD patients in the US, Agarwal et al. noted that hypertension control assessed with 44 h ABPM was achieved only in 38% of patients receiving antihypertensive drugs; however, the authors did not assess aTRH [3]. A high prevalence of aTRH ranging from 37.3 to 42.2% was reported by Li et al. in 1789 subjects undergoing peritoneal dialysis (PD) [22]. In another relatively small study of PD patients, the incidence of aTRH ranged from 27.9% to 32.9% [11].

However, the estimated prevalence of aTRH in patients with HD reported in recent studies was lower [16,18]. Tanaka et al. assessed the incidence of aTRH using pre-dialysis BP for 18% hypertensive patients (n = 2999) undergoing hemodialysis in 39 dialysis facilities in Japan participating in the Q-cohort study [16]. The authors demonstrated that aTRH is a significant independent risk factor for developing cardiovascular disease, coronary heart disease, ischemic stroke, and peripheral arterial disease. Mallamaci et al. [18] assessed the prevalence of aTRH, which was 24% among 386 treated hypertensive HD patients from 10 renal units in Europe participating in the registry of the European Renal and Cardiovascular Medicine (EURECA-m) using 44 h ABPM. It is worth emphasizing that while aTRH was considered a common problem in the HD population, most studies were conducted in pre-dialysis patients [15,23,24]. The rate of control of hypertension by drug treatment in the early studies based on pre-hemodialysis BP was variable, ranging between 30% and 70%, which resulted from the above-mentioned differences in definitions and thresholds of hypertension [2,25,26,27].

The Chronic Renal Insufficiency Cohort (CRIC) study that included 3367 hypertensive non-dialysis CKD patients revealed the high prevalence of aTRH assessed for 40.4% [15], and analysis of patients with eGFR <60 mL/min/1.73 m^2^ from two large US health care systems by An et al. estimated aTRH for 23–55% depending on the definition [24].

The aTRH incidence estimation in ESKD patients may be confounded by numerous factors, including the selected population, the method of BP estimation, and the cut-off values for uncontrolled BP.

A potential limitation of our study was using peridialytic BP measurements instead of 44h ABPM. The diagnosis of resistant hypertension requires the confirmation of poor BP control with ABPM [4,5]. Although ABPM is considered the gold standard in resistant hypertension, cut-off values for abnormal ABPM measurements differ in studies assessing ESKD patients, e.g., 130/80 mmHg [18] or 135/85 [21]. However, it should be stressed that despite the fact that among hemodialysis patients, ABPM provides reproducible readings and correlates with outcomes but is generally not used clinically, as it is cumbersome and poorly suited to day-to-day management of hypertension.

Although standardized BP measurement is recommended in CKD patients by the current KDIGO guidelines, the specific recommendations for ESKD patients on dialysis are still lacking [28]. Traditionally, HTN in HD patients has been assessed using peridialytic BP measurements taken before or after dialysis [2,26,29].

Interdialytic BP measurements, compared with peridialytic recordings, correlate better with target organ damage [30] and are a better predictor of all-cause mortality [31,32]. Therefore, experts suggest that pre- and post-dialytic BP measurements should not be the only means to diagnose or manage hypertension in patients with HD [33].

Data from a study by the European Cardiovascular and Renal Medicine (EURECA-m) working group of the ERA-EDTA published in the Sarafidis et al. study comparing pre-hemodialytic BP and 48 h ABPM in 396 HD patients revealed a similar incidence of hypertension assessed by these two techniques (89.4% versus 84.3% respectively) and a similar percentage of the patients with controlled BP (28.2% versus 28.7% respectively), but the BP status was misclassified in about 1/3 patients due to white-coat hypertension (18.2%) and that of masked hypertension (14.1%) [1]. Pre-dialysis BP 140/90 mmHg had 76% sensitivity and 54% specificity for the diagnosis of BP 130/80 mmHg by 48 h ABP monitoring with area under the ROC curve of 0.65.

However, in the analysis based on the European Renal and Cardiovascular Medicine (EURECA-m) registry, pre-dialysis BP fairly well identified TRH compared with 44 h ABPM with high sensitivity (87%) and very high specificity (99%), and the discriminatory power of pre-dialysis BP for the same diagnosis was good (area under the ROC curve: 0.93; 95% CI: 0.89–0.97) [18].

The good discriminatory value of pre-dialysis BP was explained by the authors by the fact that patients with TRH represent a subpopulation with BP values well above the hypertension thresholds of the two BP metrics (pre-dialysis BP and 44 h ABPM), and for this reason, TRH patients were less frequently misclassified by pre-dialysis BP [18]. Of note, white-coat hypertension was found just in two patients with TRH. It is worth mentioning that the definition of aTRH, according to the current guidelines, assumes that pseudoresistance cannot be excluded due to the lack of information regarding medication dose, adherence, or out-of-office BP, as in our study [4].

The use of a lower cut-off obviously increases the incidence of aTRH. Vareta et al. reported an increase in aTRH prevalence from 30% to 32.1% (for ABPM 130/80 vs. 125/75 mmHg and 27.9% to 32.9% (for office BP 140/90 vs. 130/80 mmHg) in ESKD patients treated with PD [11].

Most patients show a progressive increase in BP during the interdialytic interval and a gradual decline during the dialysis session, whereas a minority (5–15%) present a BP rise during or immediately after the dialysis session [34].

In a classical meta-analysis of Agarwal et al. [35] comparing pre-dialysis and post-dialysis BP measurements with ABPM in 18 studies, including 692 patients, authors conclude that pre-dialysis BP measurements tend to overestimate (agreements limits for systolic/diastolic BP 41.7 mmHg to −25.2 mmHg/23.7 mmHg to −18.9 mmHg), while post-dialysis measurements underestimate BP comparing ABPM values (agreement limits for systolic/diastolic BP 33.1 to −36.3/19.3 to −23.9 mmHg), although heterogeneity between BP measurements precluded pooling of the estimates [35].

Therefore, to minimize the potential discrepancies between them, we used both timepoints to assess BP to minimize the potential influence in the aTRH incidence assessment. Not surprisingly, the estimated aTRH incidence in our cohort with the use of pre-dialytic BP values was higher compared to the post-dialytic BP; however, the difference was small.

It is worth mentioning that most previous studies used only pre-dialytic BP, and in some studies, the exact timing of peridialytic BP measurement is not provided [16].

We are aware that the BP assessment in the peridialytic period in ESKD patients may encounter numerous methodological difficulties resulting from the presence of arteriovenous fistula or former multiple vascular access surgeries (preventing BP measurement on both or any arm), anxiety and discomfort during dialysis and severe vascular calcifications [28].

Studies assessing aTRH incidence among ESKD patients on HD are scarce and often performed on a limited number of patients from selected centers, often tertiary centers participating in multicenter studies. The discrepancy in the aTRH incidence in HD may result also from the differences in the population studied. Preselection bias is an important issue since aTRH occurs in a higher percentage of the population- and clinic-based samples when an at-risk group is selected and among treated hypertensive adults in clinical trials, which is likely explained by the selection of patients with demographic and comorbidity characteristics that place them at high risk for the fatal and nonfatal CVD outcomes of interest [4]. Patients from our cohort compared with those from the Tanaka et al. study had a similar age and gender distribution, dialysis vintage, and diabetes prevalence; however, they presented a higher BMI and a much higher incidence of CVD. Mean BP values were much higher (156/77 mmHg non-aTRH, 164/80 mmHg aTRH) than in our patients. Therefore, some aTRH cases might go unrecognized because patients were not prescribed ≥3 drugs at maximal doses despite uncontrolled BP [4]. It is worth emphasizing that aTRH encompasses various patient groups, such as pseudo-resistant hypertension, controlled or uncontrolled BP, and refractory hypertension (uncontrolled BP with at least five antihypertensive medication classes) [4,36].

Our cohort is similar to that of Mallamaci et al. study regarding age and gender distribution, BMI, CVD, and diabetes incidence [18]. However, the dialysis vintage was shorter than in our analysis. Of note, half of the total HD population was not included in the final analysis mainly due to the lack of ABPM, which can be the source of the bias since the criteria for performing ABPM are unclear [18]. It is worth mentioning that the authors did not count loop diuretics as a separate class of antihypertensives in contrast to our study and the Tanaka et al. study [16], which can be responsible for underestimating aTRH.

Due to residual renal function loss and anuria, diuretics have limited effectiveness in dialysis patients [37,38]. Some authors point to the fact that due to the inefficiency of diuretics in ESKD, aTRH prevalence is overestimated when counting diuretics as one class [19].

Since most studies did not include the assessment of the volume, many of the patients classified as aTRH may have had volume-mediated hypertension and no true resistance to antihypertensive treatment [11,16,18,19]. However, in our analysis, factors related to HD (dialysis vintage, dialysis time, eKt/V, ultrafiltration were not the independent risk factors of aTRH, which stays in line with the observations of the other authors assessing aTRH among HD patients [16,18]. In a subgroup of 114 HD patients, the prevalence of pre-dialysis fluid overload using the body composition monitor was 33% among aTRH patients, 34% in uncontrolled hypertensive patients, and 26% in normotensive patients [18]. Of note—67% of aTRH patients presented no fluid overload. On the other hand, fluid overload may be an important, independent factor in PD patients with aTRH [19].

Risk factors for the development of TRH in the general population of patients with HTN include older age, obesity, diabetes, smoking, increased dietary sodium intake, heart and kidney failure, and long duration of hypertension [9,39].

The strengths of our study were a high volume of unselected patients representing 30% of all dialyzed in the country according to the standard protocol using the same hardware by one of the largest European providers. To our knowledge, our cohort presents the largest one among studies assessing aTRH in ESKD treated with HD. We are fully aware that BP medication compliance is difficult to ascertain, and some assessment of medication compliance should be introduced. Prescription of hypotensive medication is feasible to assess; however, there is no possibility to cross-check the prescription with pharmacy delivery of these drugs. A body composition monitor is used to assess the volume status on a regular basis, i.e., every 12–13 weeks, to adjust dry weight and ultrafiltration rate.

The limitations are the lack of the ABPM and compliance assessment as well as some clinical features potentially increasing the risk of aTRH, including duration of hypertension, smoking habits, target organ damage, dietary sodium intake, and volume assessment.

We are aware that including both the Charlson score and some of its components (age, cardiovascular disease, and diabetes) in the model may be considered over-adjustment. However, despite the potential collinearity of the data in the model, age, cardiovascular disease, and diabetes were still statistically significant covariates.

## 6. Conclusions

Our study suggests that in the real-life world, using pre- and post-dialysis BP values, as much as 40% of HD patients may have aTRH. In ESKD patients treated with HD, aTRH seems to be multifactorial, influenced by patient-related factors rather than dialysis-related factors. Various definitions of aTRH preclude easy comparisons between studies. We could suspect that if the chronic HD units ensured medication compliance and fully lowered fluid volume, the prevalence of resistant hypertension would be very much less than the 38–40% found in our population, and maybe it contributes to lowered all-cause and/or cardiovascular mortality.

## Figures and Tables

**Table 1:** Characteristics of all the patients; normotensive (NT) and hypertensive (HTN) groups according to the pre-dialysis (1) and post-dialysis (2) blood pressure criteria.

	All pts.	NT^1^	HTN^1^	NT^2^	HTN^2^
	N = 5879	N = 549	N = 5330	N = 639	N = 5240
Age [years]	65.2 (14.2)	65.8 (15.2)	65.2 (14.1)	65.8 (14.6)	65.2 (14.2)
Gender [males]	3519 (59.9%)	289 (52.6%)	3230 (60.6%)	352 (55.1%)	3167 (60.4%)
BMI [kg/m^2^]	27.5 (6.02)	26.5 (5.90)	27.6 (6.02)	26.8 (5.87)	27.6 (6.03)
Charlson score	4.17 (1.90)	3.60 (1.88)	4.23 (1.90)	3.59 (1.83)	4.24 (1.90)
CVD	3564 (60.6%)	231 (42.1%)	3333 (62.5%)	266 (41.6%)	3298 (62.9%)
CHF	2114 (36.0%)	124 (22.6%)	1990 (37.3%)	144 (22.5%)	1970 (37.6%)
CAD	1678 (28.5%)	99 (18.0%)	1579 (29.6%)	112 (17.5%)	1566 (29.9%)
PAD	1708 (29.1%)	121 (22.0%)	1587 (29.8%)	132 (20.7%)	1576 (30.1%)
Cerebrovascular	754 (12.8%)	37 (6.74%)	717 (13.5%)	49 (7.67%)	705 (13.5%)
DM	2068 (35.2%)	132 (24.0%)	1936 (36.3%)	154 (24.1%)	1914 (36.5%)
Dialysis vintage [months]	59.6 (64.0)	49.0 (70.9)	60.5 (63.3)	50.9 (69.5)	60.4 (63.3)
Dialysis time [min/week]	702 (115)	642 (177)	708 (105)	639 (179)	709 (102)
eKt/V	1.37 (0.31)	1.36 (0.36)	1.37 (0.31)	1.37 (0.41)	1.37 (0.30)
UF [mL]	2069 (914)	1681 (897)	2109 (906)	1762 (926)	2107 (905)
Na [mmol/L]	139 (5.02)	139 (5.5)	139 (5.0)	139 (5.4)	139 (5.0)
No. of BP drugs	2.33 (1.51)	0.00 (0.00)	2.57 (1.37)	0.00 (0.00)	2.61 (1.34)
Pre-SBP [mmHg]	141 (17.0)	125 (11.9)	142 (16.6)	129 (14.9)	142 (16.8)
Pre-DBP [mmHg]	76.2 (9.71)	71.6 (7.98)	76.7 (9.75)	73.7 (8.89)	76.5 (9.77)
Pre-HR [1/min]	74.0 (8.51)	75.8 (8.62)	73.8 (8.47)	75.9 (8.16)	73.8 (8.52)
Post-SBP [mmHg]	136 (17.7)	123 (15.5)	138 (17.4)	122 (12.7)	138 (17.5)
Post-DBP [mmHg]	74.8 (9.19)	70.9 (8.43)	75.2 (9.17)	70.8 (7.81)	75.3 (9.23)
Post-HR [1/min]	71.9 (8.65)	74.4 (9.79)	71.7 (8.49)	74.3 (9.28)	71.6 (8.53)

NT^1^—normotensive according to pre-dialysis criteria, NT^2^—normotensive according to post-dialysis criteria, HTN^1^—hypertensive to pre-dialysis criteria, HTN^2^—hypertensive to post-dialysis criteria, BMI—body mass index, Charlson—Charlson comorbidity index score, CVD—cardiovascular disease, CHF—congestive heart failure, CAD—coronary artery disease, PAD—peripheral artery disease, cerebrovascular—cerebrovascular disease, DM—diabetes mellitus, dialysis time—effective weekly dialysis time, eKt/V—estimated dialysis adequacy marker when K means dialyzer clearance of urea; t—dialysis time, V—volume, UF—ultrafiltration, Na—pre-dialysis plasma sodium concentration, BP—blood pressure, pre—pre-dialysis, post—post-dialysis, SBP—systolic blood pressure, DBP—diastolic blood pressure, HR—heart rate.

**Table 2:** Characteristics of the apparent resistant hypertension (aTRH) and non-apparent resistant hypertension (non-aTRH) groups according to the pre-dialysis (1) and post-dialysis (2) blood pressure criteria.

	aTRH^1^	Non-aTRH^1^	aTRH^2^	Non-aTRH^2^	*p* ^1^	*p* ^2^
	N = 2179	N = 3151	N = 2014	N = 3226		
Age [years]	63.6 (13.8)	66.3 (14.2)	63.7 (14.1)	66.0 (14.1)	<0.001	<0.001
Gender [Males]	1369 (62.8%)	1861 (59.1%)	1252 (62.2%)	1915 (59.4%)	0.006	0.047
BMI [kg/m^2^]	28.1 (6.14)	27.3 (5.90)	27.9 (6.10)	27.4 (5.98)	<0.001	0.002
Charlson score	4.32 (1.87)	4.16 (1.91)	4.32 (1.87)	4.19 (1.92)	0.003	0.012
CVD	1418 (65.1%)	1915 (60.8%)	1318 (65.4%)	1980 (61.4%)	0.002	0.003
CHF	861 (39.5%)	1129 (35.8%)	804 (39.9%)	1166 (36.1%)	0.007	0.007
CAD	671 (30.8%)	908 (28.8%)	620 (30.8%)	946 (29.3%)	0.127	0.275
PAD	668 (30.7%)	919 (29.2%)	622 (30.9%)	954 (29.6%)	0.254	0.329
Cerebrovascular	321 (14.7%)	396 (12.6%)	305 (15.1%)	400 (12.4%)	0.025	0.005
DM	892 (40.9%)	1044 (33.1%)	826 (41.0%)	1088 (33.7%)	<0.001	<0.001
Dialysis vintage [months]	54.9 (55.4)	64.4 (68.1)	53.9 (53.0)	64.6 (68.9)	<0.001	<0.001
Dialysis time [min/week]	719 (89.3)	700 (114)	717 (89.8)	704 (109)	<0.001	<0.001
eKt/V	1.35 (0.28)	1.38 (0.32)	1.36 (0.29)	1.38 (0.31)	<0.001	0.016
UF [ml]	2287 (889)	1986 (897)	2250 (891)	2017 (902)	<0.001	<0.001
Pre-Na [mmol/L]	139 (5.1)	139 (4.9)	139 (5.0)	139 (5.4)	0.858	0.683
No. of BP drugs	3.86 (0.75)	1.67 (0.92)	3.93 (0.74)	1.79 (0.91)	0.000	0.000
Pre-SBP [mmHg]	150 (14.5)	137 (16.1)	149 (15.3)	138 (16.2)	<0.001	<0.001
Pre-DBP [mmHg]	78.7 (9.72)	75.2 (9.53)	78.3 (10.1)	75.4 (9.42)	<0.001	<0.001
Pre-HR [1/min]	73.0 (8.61)	74.4 (8.33)	72.7 (8.71)	74.4 (8.34)	<0.001	<0.001
Post-SBP [mmHg]	145 (15.6)	133 (16.9)	147 (15.2)	133 (16.6)	<0.001	<0.001
Post-DBP [mmHg]	77.6 (8.60)	73.6 (9.20)	78.0 (8.75)	73.6 (9.12)	<0.001	<0.001
Post-HR [1/min]	70.7 (8.31)	72.4 (8.54)	70.5 (8.50)	72.3 (8.47)	<0.001	<0.001

BMI—body mass index, Charlson—Charlson comorbidity index score, CVD—cardiovascular disease, CHF—congestive heart failure, CAD—coronary artery disease, PAD—peripheral artery disease, cerebrovascular—cerebrovascular disease, DM—diabetes mellitus, Na—pre-dialysis plasma sodium concentration, BP—blood pressure, dialysis time—effective weekly dialysis time, eKt/V, UF—ultrafiltration, pre—pre-dialysis, post—post-dialysis, SBP—systolic blood pressure, DBP—diastolic blood pressure, HR—heart rate.

**Table 3:** Antihypertensive medications used in the apparent resistant hypertension (aTRH) and non-apparent resistant hypertension (non-aTRH) groups according to the pre-dialysis (1) and post-dialysis (2) blood pressure criteria.

	aTRH^1^	Non-aTRH^1^	aTRH^2^	Non-aTRH^2^	*p* ^1^	*p* ^2^
	N = 2179	N = 3151	N = 2014	N = 3226		
ACEi	983 (45.1%)	480 (15.2%)	938 (46.6%)	525 (16.3%)	<0.001	<0.001
ARB	386 (17.7%)	152 (4.82%)	371 (18.4%)	167 (5.18%)	<0.001	<0.001
CCB	1858 (85.3%)	793 (25.2%)	1748 (86.8%)	903 (28.0%)	<0.001	<0.001
BB	1910 (87.7%)	1915 (60.8%)	1767 (87.7%)	2058 (63.8%)	<0.001	<0.001
Loop diuretic	1688 (77.5%)	1512 (48.0%)	1577 (78.3%)	1623 (50.3%)	<0.001	<0.001
Other	1573 (72.2%)	415 (13.2%)	1504 (74.7%)	484 (15.0%)	<0.001	<0.001

ACEi—angiotensin converting enzyme inhibitor, ARB—angiotensin II receptor blockers, CCB—calcium channel blockers, BB—beta blockers.

**Table 4:** Unadjusted and adjusted odds ratios (OR) with 95%CI for aTRH risk diagnosed according to pre-dialysis and post-dialysis blood pressure criteria.

	Pre-Dialysis		Post-Dialysis	
	OR Unadjusted	OR Adjusted	OR Unadjusted	OR Adjusted
Age	0.99 [0.98–0.99]	0.99 [0.98–0.99]	0.99 [0.98–0.99]	0.99 [0.98–0.99]
Gender [Males]	1.17 [1.05–1.31]	0.94 [0.83–1.07]	1.12 [1.00–1.26]	0.96 [0.84–1.09]
BMI	1.02 [1.01–1.03]	1.01 [1.00–1.03]	1.01 [1.01–1.02]	1.00 [0.99–1.01]
Charlson score	1.04 [1.01–1.07]	0.99 [0.95–1.04]	1.04 [1.01–1.07]	0.98 [0.94–1.03]
CVD	1.20 [1.07–1.35]	1.26 [1.08–1.46]	1.19 [1.06–1.34]	1.30 [1.12–1.51]
DM	1.40 [1.25–1.57]	1.26 [1.08–1.47]	1.37 [1.22–1.53]	1.28 [1.09–1.49]
Dialysis vintage	1.00 [1.00–1.00]	1.00 [1.00–1.00]	1.00 [1.00–1.00]	1.00 [1.00–1.00]
Dialysis time	1.00 [1.00–1.00]	1.00 [1.00–1.00]	1.00 [1.00–1.00]	1.00 [1.00–1.00]
eKt/V	0.68 [0.56–0.82]	0.81 [0.65–1.01]	0.79 [0.65–0.96]	0.93 [0.75–1.16]
UF	1.00 [1.00–1.00]	1.00 [1.00–1.00]	1.00 [1.00–1.00]	1.00 [1.00–1.00]

BMI—body mass index, Charlson—Charlson comorbidity index score, CVD—cardiovascular disease, DM—diabetes mellitus, dialysis time—effective weekly dialysis time, eKt/V, UF—ultrafiltration.

## Data Availability

The data underlying this article will be shared on reasonable request to the corresponding author.
